# Identification of Sare0718 As an Alanine-Activating Adenylation Domain in Marine Actinomycete *Salinispora arenicola* CNS-205

**DOI:** 10.1371/journal.pone.0037487

**Published:** 2012-05-24

**Authors:** Sisi Xia, Yanlin Ma, Wei Zhang, Yi Yang, Shaowen Wu, Minzhe Zhu, Lingfu Deng, Bing Li, Zhonglai Liu, Chao Qi

**Affiliations:** 1 Hubei Key Laboratory of Genetic Regulation & Integrative Biology, College of Life Science, Central China Normal University, Wuhan, China; 2 The Fourth Hospital of Hebei Medical University, Shijiazhuang, P. R. China; Semmelweis University, Hungary

## Abstract

**Background:**

Amino acid adenylation domains (A domains) are critical enzymes that dictate the identity of the amino acid building blocks to be incorporated during nonribosomal peptide (NRP) biosynthesis. NRPs represent a large group of valuable natural products that are widely applied in medicine, agriculture, and biochemical research. *Salinispora arenicola* CNS-205 is a representative strain of the first discovered obligate marine actinomycete genus, whose genome harbors a large number of cryptic secondary metabolite gene clusters.

**Methodology/Principal Findings:**

In order to investigate cryptic NRP-related metabolites in *S. arenicola* CNS-205, we cloned and identified the putative gene *sare0718* annotated “amino acid adenylation domain”. Firstly, the general features and possible functions of *sare0718* were predicted by bioinformatics analysis, which suggested that Sare0718 is a soluble protein with an AMP-binding domain contained in the sequence and its cognate substrate is L-Val. Then, a GST-tagged fusion protein was expressed and purified to further explore the exact adenylation activity of Sare0718 *in vitro*. By a newly mentioned nonradioactive malachite green colorimetric assay, we found that L-Ala but not L-Val is the actual activated amino acid substrate and the basic kinetic parameters of Sare0718 for it are *K_m_* = 0.1164±0.0159 (mM), *V_max_* = 3.1484±0.1278 (µM/min), *k_cat_* = 12.5936±0.5112 (min^−1^).

**Conclusions/Significance:**

By revealing the biochemical role of *sare0718* gene, we identified an alanine-activating adenylation domain in marine actinomycete *Salinispora arenicola* CNS-205, which would provide useful information for next isolation and function elucidation of the whole cryptic nonribosomal peptide synthetase (NRPS)-related gene cluster covering Sare0718. And meanwhile, this work also enriched the biochemical data of A domain substrate specificity in newly discovered marine actinomycete NRPS system, which bioinformatics prediction will largely depend on.

## Introduction

Actinomycetes are a remarkably prolific source of structurally diverse natural products, including many that possess pharmaceutically relevant biological activities [1]. The search for new effective microbial natural products, however, has long been hampered by increasing drug-resistant pathogens as well as growing rediscovery of known antibiotics from terrestrial actinobacteria [2]. As a response, efforts are shifted to the broadly-untapped ocean [3]. Owing to the particular living conditions of heavy salinity, high pressure, low oxygen and short nutrient, marine actinomycetes have developed specific metabolic adaptations to their ecological environment and therefore could deliver a series of structurally diverse secondary metabolites, which renders them a promising treasure for the discovery of new natural products [4], [5].

Nonribosomal peptides (NRPs), with complicated structures and diverse bioactivities, usually 3–15 amino acids in length, represent a large group of valuable natural products that are widely applied in medicine, agriculture, and biochemical research [6], [7]. Important NRPs include vancomycin (antibiotic) [8], bleomycin (antitumor agent) [9], cyclosporine (immunosuppressant) [10], enterobactin (siderophore) [11], surfactin A (biosurfactant) [12], syringomycin (phytotoxin) [13], and so on. The biosynthesis of NRPs is performed on nonribosomal peptide synthetases (NRPSs), a large multienzyme complex that can carry out up to several dozen reactions in a coordinated manner. NRPSs are composed of modules, each capable of carrying out one cycle of chain extension. A minimal elongation module harbors three core catalytic domains — the adenylation (A), peptidyl carrier protein (PCP or T), and condensation (C) domains, necessary for recognition, activation, and covalent binding of a single building block monomer, as well as for peptide-bond formation with the growing chain [14]. Obviously, the modular nature of NRPSs provides an attractive opportunity for rational design of recombinant organisms to produce novel ‘unnatural’ natural products by combinatorial biosynthesis or total *in vitro* (bio)synthesis, the application of which relies highly on elucidations of numerous NRPS gene clusters and their biosynthetic pathways [15], [16], [17]. Particularly, among the three essential catalytic domains, A domains are responsible for the selection and activation of cognate substrates; they are critical in dictating the identity of the amino acid building blocks to be incorporated during nonribosomal peptide (NRP) biosynthesis. Therefore, the composition and structural diversity of NRPs are derived primarily from the building block-activating A domains in each NRPS module.

One of the most significant milestones in marine microbiology was the report and confirmation of the first seawater-dependent actinomycete genus ‘*Salinispora*’ in 2005 [18], from which a number of unprecedented drug-like compounds were separated [19], such as the potent proteasome inhibitor salinosporamide A that has currently entered clinical trials for cancer treatment [20]. *Salinispora arenicola* CNS-205, isolated from Palau's deep-sea sediments, is a representative strain of this genus. Its genome sequencing (GenBank accession no. CP000850) in 2007 revealed 10 NRPS-related biosynthetic gene clusters [21], [22], whereas only one NRPS gene cluster's products — cyclomarins and cyclomarazines were detected in the fermentation broth [19], [23]. This observation means that the *S. arenicola* chromosome harbors a number of cryptic NRPS-related gene clusters, whose “orphan pathways” and metabolites await us to reveal and annotate.

For the genome of *S. arenicola* CNS-205, 16 genes annotated “amino acid adenylation domain” can be retrieved by BLAST on NCBI (http://www.ncbi.nlm.nih.gov). Interestingly, except for *sare0718*, each of the 15 other “amino acid adenylation domain” loci has a corresponding NRPS gene cluster (as shown in Table 1) and all of them belong to the 10 NRPS-related biosynthetic gene clusters predicted by Penn K etc. in 2009 using bioinformatics [21], [22]. In contrast, the definite function of *sare0718* or its NRPS-related gene cluster remains poorly understood. Consequently, we expressed *sare0718* gene and identified its biochemical roles as an alanine-activating adenylation domain. Our work would facilitate next isolation and functional elucidation of the whole cryptic NRPS-related gene cluster containing *sare0718*.

**Table 1:** 15 predicted A domain loci and corresponding NRPS gene cluster in *Salinispora arenicola* CNS-205.

Adenylation domains location	Corresponding NRPS gene cluster name*	Cluster location	Actual or predicted product
Sare0353 Sare0357 Sare0362 Sare0363	SA *nrps1*	Sare0345–0367	Pentapeptide
Sare2071	SA *sid1*	Sare2070–2081	Yersiniabactin-like siderophore
Sare2407	SA *pksnrps2*	Sare2400–2409	PK-NRP hybrid
Sare2948 Sare2961 Sare2962	SA *nrps2*	Sare2939–2968	Tetrapeptide
Sare3052	SA *nrps3*	Sare3051–3063	Dipeptide
Sare4562	SA *cym*	Sare4547–4569	Cyclomarin
Sare4890 Sare4891 Sare4894 Sare4895	SA *nrps4*	Sare4885–4904	Tetrapeptide

* Data from K Penn *et al* [21], [22].

## Results

### Bioinformatics analysis of *sare0718* gene

Before performing the experiment, bioinformatics analysis of *sare0718* was carried out to better understand the general features and possible functions of the gene. By analysis of Protparam, Protscale and other programs on the ExPASy server [24], we obtained some detailed information about Sare0718 protein (Accession no. YP_001535628): molecular weight at 60.287 kDa, pI at 6.44, and hydrophobicity score between −1.710∼1.557 by which Sare0718 is judged as a hydrophilic protein. Prediction by program TMHMM Server v. 2.0 [25] and SignalP 4.0 [26] suggested that neither a signal peptide nor transmembrane region is included in the protein. Besides, an AMP-binding domain (between 89–478 amino acid residues) is found in Sare0718 by SMART [27]. The specificity-conferring code analysis by the online website “PKS/NRPS Analysis Web-site” showed that the signature sequence constituting the substrate-binding pocket of Sare0718 was DMWIAAAIVK and its cognate substrate was L-Val [28]. Finally, with DNAMAN software, we compared the protein homology between Sare0718 and each of the eight terrestrial-derived alanine-activating adenylation domains posted in PKS-NRPS database [29]. As can be seen in Table 2, the protein similarity between Sare0718 and them is just 18.18%∼25.83%.

**Table 2:** Protein homology analysis by sequence alignment.

Adenylation domains	Producer organisms	Protein homology with Sare0718 (%)
Bleomycin 005-A1	*Streptomyces verticillus*	25.83
Bleomycin 009-A1	*Streptomyces verticillus*	23.4
Microcystin 001-A2	*Microcystis aeruginosa*	24.26
HCtoxin 001-A2	*Cochliobolus carbonum*	20
HCtoxin 001-A3	*Cochliobolus carbonum*	20.26
Fengycin 005-A2	*Bacillus subtilis*	18.18
Cyclosporin 001-A1	*Tolypocladium inflatum*	21.75
Cyclosporin 001-A11	*Tolypocladium inflatum*	23.58

### Cloning, expression, purification and western blotting analysis of Sare0718 fusion protein

**Figure 1:**
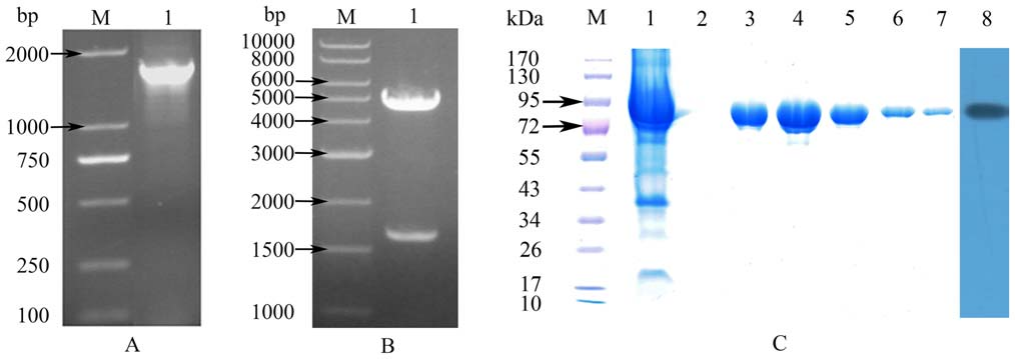
Molecule cloning and protein expression of *Sare0718*. (A) Amplification of *sare0718* gene by PCR. M: DL2000 DNA marker; 1: PCR product of *sare0718*. (B) Enzyme digestion identification of pGEX-2T-*sare0718* recombinant plasmid. M: 1 kb DNA ladder; 1: pGEX-2T-*sare0718* digested with *BamH* I and *EcoR* I. (C) 10% SDS-PAGE and Western Blotting analysis of recombinant Sare0718 purified by Glutathione Sepharose4B affinity chromatography column. M: Protein marker; 1: induced *E.coli* BL21(DE3) transformed with pGEX-2T-*sare0718*; 2–7: elution samples with 20 mM GSH(1 ml/tube); 8: Western Blotting image of purified recombinant Sare0718.

The gene *sare0718* was amplified by PCR from *S. arenicola* CNS-205 genomic DNA. Analysis of 1% agrose gel electrophoresis (Figure 1A) showed that the size of *sare0718* gene is about 1700 bp, similar to the reported length (1671 bp). To construct a prokaryotic expression plasmid, PCR product of *sare0718* was cloned into pGEX-2T vector. Restriction enzyme (*BamH* I/*EcoR* I) digestion of the recombinant plasmid resulted in two DNA fragments of expected size (Figure 1B). Finally, the resulting expression constructs were further verified by direct DNA sequencing, and designated pGEX-2T-*sare0718*.

*E.Coli* BL21 (DE3) competent cells were then transformed with recombinant plasmid pGEX-2T-*sare0718*, and induced with 0.2 mM IPTG at 16°C for 24 h to express target protein. After sonification of the induced *E. coli* cells, soluble recombinant protein in the supernatant was purified by Glutathione-Sepharose4B affinity chromatography, followed by desalting treatment for next enzyme assay. As can be seen in the 10% SDS-PAGE gel (Figure 1C: lane 1–7), Sare0718 (∼61 kDa) was expressed in *E.Coli* BL21 (DE3) as a GST-tagged fusion protein of about 87 kDa, consistent with the predicted molecular mass (here, GST-tag: ∼26 kDa). By program BandScan 5.0 and BCA Protein Assay Reagent, protein purity of ∼95% (Figure 1C: lane 6) and protein yield of 4∼5 mg/L were determined, respectively. Besides, the presence of an N-terminal GST-tag in Sare0718 recombinant protein was verified by Western blotting (Figure 1C: lane 8).

### Malachite green colorimetric assay for Sare0718 enzyme activity

On NRP assembly line, A domains are responsible for the following biochemical reaction: Amino acid+ATP→Aminoacyl-AMP+PPi. In this study, a nonradioactive high-throughtout malachite green colorimetric assay [30] was used to measure Sare0718 enzyme activity. Inorganic pyrophosphatase was coupled in the assay to convert the PPi produced during aminoacyl-AMP formation to orthophosphate (Pi), followed by a molybdate/malachite green reagent to measure Pi concentrations. Enzyme assays were performed in 96-well plates at 25°C. Detailed reaction system and process were designed as described before [31], [32].

#### Determination of Sare0718 substrate specificity

**Figure 2:**
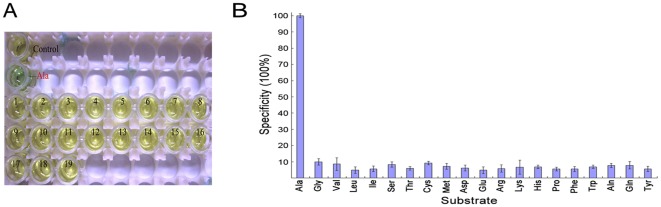
Determination of Sare0718 substrate specificity among the 20 proteinogenic amino acids. (A) Schematic representation of the substrate screening by malachite green-ammonium molybdate colorimetric assay. Control: the reaction system without Sare0718 (compared with the below Ala sample well); Well 1–19: reaction systems with the substrate of Gly, Val, Leu, Ile, Ser, Thr, Cys, Met, Asp, Glu, Arg, Lys, His, Pro, Phe, Trp, Asn, Gln and Tyr, respectively. (B) Relative activities (substrate specificity) of recombinant Sare0718 for 20 proteinogenic amino acids. Individual substrate activities are presented by bars and the highest activity defined as 100%.

All 20 proteinogenic amino acids were tested to investigate the substrate specificity of Sare0718. As can be seen in Figure 2A, compared with the control well (containing all the reactants added to the L-alanine sample well, except that Sare0718 was omitted), the adenylation of L-alanine by Sare0718 led to a remarkable color conversion of malachite green reagent (from pale yellow-green to blue-green) whereas the other 19 proteinogenic amino acids couldn't make such an obvious color change. Furthermore, the relative activities of Sare0718 for different amino acids (Figure 2B) also showed that, of the 20 proteinogenic amino acids, L-alanine was the specific substrate of Sare0718.

#### Determination of kinetic parameters (*K_m_*, *V_max_*, and *k_cat_*) under the optimum incubation time and enzyme concentration

**Figure 3:**
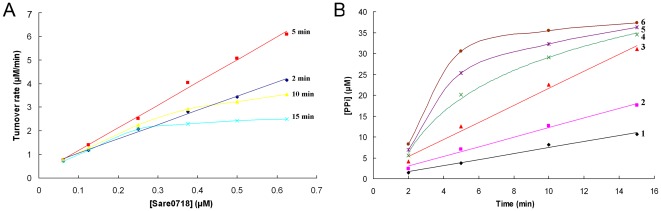
Selection of optimum incubation time and enzyme concentration for kinetic parameter assay. To determine the scope of initial reaction velocity, enzymatic reactions (100 µl) containing 0.5 mM alanine, 0.5 mM ATP and different concentrations of purified Sare0718 were performed at 25°C in 96-well plates for various times (2 min, 5 min, 10 min, and 15 min). (A) Enzyme concentration curve: reaction velocity versus concentration of Sare0718. (B) Time course curve: concentration of production PPi versus reaction time. Sare0718 concentrations in curve 1–6 were 0.0625 µM, 0.125 µM, 0.25 µM, 0.375 µM, 0.5 µM and 0.625 µM, respectively.

**Figure 4:**
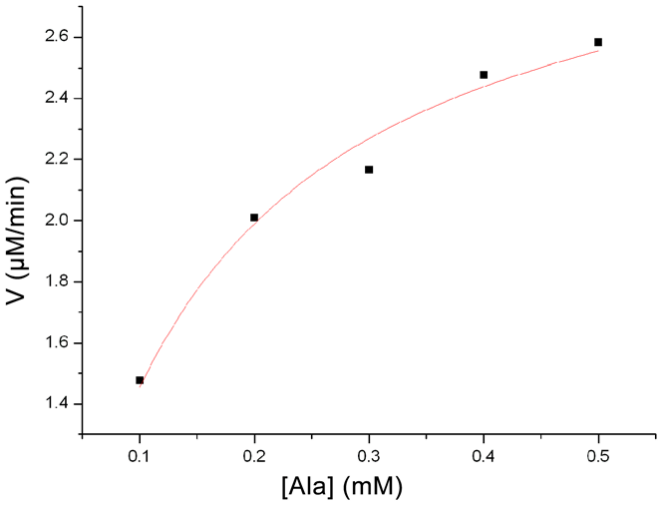
Determination of kinetic parameters of Sare0718 for L-Ala by Michaelis-Menten analysis using nonlinear regression.

In order to ensure that enzyme assays for kinetic parameters were carried out within the scope of initial reaction velocity, optimum incubation time and enzyme concentration had to be firstly determined. With various concentrations of Sare0718 (0.0625 µM, 0.125 µM, 0.25 µM, 0.375 µM, 0.5 µM, and 0.625 µM), enzymatic reactions were performed in 96-well plates at 25°C for different times (2 min, 5 min, 10 min, and 15 min). Using the PPi formation velocity as a function of Sare0718 concentration, we obtained an enzyme concentration curve (Figure 3A). It showed that within 5 min, reaction velocity kept a positive linear relationship with Sare0718 concentration and reached its maximum value. On the other hand, the time course curve (Figure 3B) was also plotted by using [PPi] (PPi concentration) as a function of reaction time, which suggested that PPi concentration remained a positive linear relationship with reaction time when Sare0718 concentration was within 0.25 µM. Taken together, the optimum incubation time of 5 min and enzyme concentration of 0.25 µM were chosen for downstream kinetic assays. Then, under Sare0718 cencentration of 0.25 µM and incubation time of 5 min, kinetic parameters (*K_m_* and *V_max_*) can be determined by varying the amount of one substrate (alanine: 0.1 mM, 0.2 mM, 0.3 mM, 0.4 mM, 0.5 mM) and keeping the other reactants (ATP: 0.5 mM) at a saturated concentration. By Michaelis-Menten analysis using nonlinear regression (V versus [Ala], see Figure 4), the kinetic parameters of Sare0718 for L-alanine were determined as followings: *K_m_* = 0.1164±0.0159 (mM), *V_max_* = 3.1484±0.1278 (µM/min), *k_cat_* = 12.5936±0.5112 (min^−1^).

## Discussion

Among the three core components of NRPS elongation modules, an amino acid adenylation domain (about 550 aa in size) catalyzes the first step of each reaction cycle, responsible for selecting a specific amino acid substrate and activating it as the aminoacyl-AMP under the consumption of ATP [14]. Moreover, a typical NRPS contains one A domain for each building block present in the resulting organic molecule [15]. Thus, A domains play a key role in NRP biosynthesis — the composition and structural diversity of NRPs are derived primarily from the building block-activating A domains in each NRPS module. For identification of NRPS gene clusters, A domains are often used as screening markers and gene probes to assess the capability of NRP production in detected microbes or to clone the whole target gene cluster [33].

In this paper, *sare0718*, the gene of a putative amino acid adenylation domain, was cloned and identified as an alanine-activating adenylation domain for further investigation into cryptic NRPS-related gene clusters in *S. arenicola* CNS-205. Results presented here suggested that Sare0718 could *in vitro* specially recognize and activate L-alanine under the consumption of ATP. First of all, the general features and possible functions of *sare0718* gene were predicted by bioinformatics analysis before our experiments. Data indicated that Sare0718 is a soluble protein without signal peptide and transmembrane domain. The analysis of conserved domain revealed that an AMP-binding domain is contained within Sare0718 sequence at the 89–478 amino acid residues. Based on these bioinformatics data, experiments were performed to study the concrete function of *sare0718* gene.

The traditional method for analyzing A domain activity is the ATP-[^32^P] pyrophosphate (PPi) exchange assay, which has been restricted to follow the formation of isotopically labeled ATP from aminoacyl-AMP reacted with an excess of [^32^P] PPi [34]. As a result, rapid and high-throughput substrate-screening of A domains by this method is usually hindered by cumbersome and significant handling of radioactive materials. Fortunately, in 2009 Thomas J. McQuade reported a nonradioactive high-throughtout colorimetric assay for screening and characterization of A domains, in which inorganic pyrophosphatases were used to convert the PPi produced during aminoacyl-AMP formation to orthophosphate (Pi) with a molybdate/malachite green reagent to measure Pi concentrations [30]. This method is not only accurate and robust, but also can be performed in 96- or 384-well plate formats. In our work, with the 20 proteinogenic amino acids as detecting substrates, this facile colorimetric assay was used in 96-well plate to analyze the enzymatic activity of recombinant Sare0718 protein. The results showed that Sare0718 was specific to L-alanine over the other proteinogenic amino acids and its basic kinetic parameters for L-alanine were determined as follows: *K_m_* = 0.1164±0.0159 (mM), *k_cat_* = 12.5936±0.5112 (min^−1^). Therefore, we have acquired an active alanine-activating adenylation domain.

With A domains being primary determinants of NRPS module selectivity during NRP assembly, the research on A domain substrate specificity becomes the key point for realizing NRP combinatorial biosynthesis and has long been the common concern of scholars around the world. Based on the first solved crystal structure of a representative phenylalanine-activating A domain in complex with its two substrates in 1997 [35], the specificity-conferring codes of NRPS A domains were deciphered by Stachelhaus and coworkers [36] in 1999, and recently further refined by Rausch and coworkers [37]. These bioinformatics studies have greatly promoted the substrate prediction and research on A domains. Nevertheless, examples of codes for which no substrate could be matched or the predicted substrate specificity does not correspond to the actual activated amino acid have been reported [38], [39]. In this work, Sare0718 is just such a special case. Before measuring enzymatic activity of Sare0718, we had analyzed its code by the online website “PKS/NRPS Analysis Web-site” [28], which showed that the signature sequence constituting the substrate-binding pocket of Sare0718 was DMWIAAAIVK and its cognate substrate was L-Val. However, data presented in this paper indicated that the actual recognized amino acid for Sare0718 was L-Ala. Similarly, bioinformatics analysis of the seven A domains in CymA (a NRPS gene cluster encoding cyclomarins and cyclomarazines in *S. arenicola* CNS-205) only accurately correlated module-4 to the activation of L-phenylalanine, while the specificities of the remaining A domains were less clear [23]. In addition, during the elucidation of thiocoraline gene cluster in *Micromonospora sp*, the same situation occurred, in which the NRP metabolite was coincidentally also produced by a marine-derived actinomycete [38]. As to the special examples mentioned above, we speculate that these results might be partly due to the remarkable differences in physiology and phylogenesis between marine actinomycetes and their terrestrial relatives. In fact, there were not any true marine actinomycetes that had been reported and identified [18], [22] when Torsten proposed specificity-conferring selection rules in 1999 [36], so the experimental data on which they established the NRPS code system were all derived from terrestrial microorganisms. On the other hand, the low protein homology between Sare0718 and some other terrestrial-derived alanine-activating adenylation domains (18.18%∼25.83%, see Table 2) also supported the speculation. Taken together, these observations indicate that the marine actinomycete NRPSs, emerging as a newly discovered system, still need a large number of biochemical characterization data to facilitate and improve bioinformatics research on a more accurate forecast of A domain substrate specificity.

Currently, “amino acid adenylation domains” are mainly found as components of NRPSs or hybrid NRPSs (NRPS/FASs or NRPS/PKSs etc). As for S. *arenicola* CNS-205, 10 NRPS-related gene clusters were revealed in its 5 786 361 bp circular chromosome by completed genome sequencing analysis [23]. However, according to our analysis in Table 1, gene *sare0718* belongs to none of them. Besides, we found that the nearest genes adjacent to *sare0718* are *sare0716* (Enoyl-CoA hydratase/isomerase), *sare0717* (a hypothetical protein), and *sare0719* (4′-phosphopantetheinyl transferase). It means that, Sare0718, homologous to “amino acid adenylation domains” of NRPSs, was identified by our experimental data as an alanine-activating adenylation domain but not integrated into a typical NRPS module. In classical linear NRPSs, the three core catalytic domains are integrated in an order of “C-A-PCP” into each elongation module. But during the past two decades, sequencing efforts have revealed many nonlinear biosynthetic clusters that deviate from the standard linear ones in their domain organization and biosynthetic logic. Stand-alone C, A or PCP domains participating in the nonlinear biosynthesis have been reported [9], [40], [41], [42]. For instance, the enzyme NovL involved in the biosynthesis of the antibiotic novobiocin from *Streptomyces spheroides* [41], [42] is an impressive example of a stand-alone A domain for unusual peptide antibiotic biosynthesis. Here, the stand-alone Sare0718 still needs prospective experiments to investigate its in-depth function *in vivo*.

In summary, with the 20 proteinogenic amino acids as detecting substrates, we have *in vitro* identified Sare0718 in *S. areniocola* CNS-205 as an alanine-activating adenylation domain by a nonradioactive malachite green colorimetric assay. It is still worth mentioning that some untested rare amino acids or even a structurally related but unidentified compound might be also activated by Sare0718 since NRPS A domains can often accept non-proteinogenic amino acids, and the *in vivo* substrate may not be L-alanine. Our investigation into the substrate specificity of Sare0718 as described in this paper is useful information in identifying the cryptic NRPS-related gene cluster containing *sare0718*. Meanwhile, it also enriched the biochemical data of A domain substrate specificity in newly discovered marine actinomycete NRPS system, which bioinformatics prediction will largely depend on.

## Materials and Methods

### Bioinformatics analysis of *Sare0718* gene

In order to aid in understanding of the *sare0718* gene, certain computational methods were utilized. First of all, molecular weight, pI and hydrophobicity of Sare0718 were analyzed by Protparam, Protscale and other programs on the ExPASy server [24]. Transmembrane region, signal peptide and conserved domain were predicted by program TMHMM Server v. 2.0 [25], SignalP 4.0 [26] and SMART [27], respectively. Moreover, the specificity-conferring code and cognate substrate of Sare0718 were analyzed by the online website “PKS/NRPS Analysis Web-site” [28]. Finally, DNAMAN software (http://www.lynnon.com/) was used to compare the protein homology between Sare0718 and each of the eight terrestrial-derived alanine-activating adenylation domains posted in PKS-NRPS database [29].

### Cloning, expression, purification and western blotting analysis of Sare0718 fusion protein

#### Construction of pGEX-2T-*sare0718* expression plasmid

The gene *sare0718* was amplified by PCR from genomic DNA isolated from the producer organism *S. arenicola* CNS-205 (GenBank accession no. CP000850). The forward primer (5′- CCGGGATCCCAGCGCACCTCGACCACCACGACGGC-3′) and reverse primer (5′-CCCGAATTCTCGCGTCGCCCCCTGCTCCTGGCTG-3′) used for amplification of the *sare0718* gene introduced *BamH* I and *EcoR* I restriction sites (underlined), respectively. Polymerase chain reaction (PCR) was carried out using Phusion DNA polymerase (New England Biolabs, cat.no. F-530S) as indicated by the manufacturer. The PCR product of *sare0718* was cloned into the digested pGEX-2T vector using the corresponding *BamH* I/*EcoR* I restriction sites. The recombinant plasmid bearing DNA encoding Sare0718 protein was verified by sequencing and designated pGEX-2T-*sare0718*.

#### Expression, purification and western blotting analysis of Sare0718 fusion protein

For recombinant protein expression and purification, *E.coli* BL21 (DE3) competent cells were transformed with purified pGEX-2T-sare0718 plasmids. Fresh transformants harboring the Sare0718 construct were grown at 37°C in Luria-Bertani (LB) medium (400 ml started with 1% inoculums from a 10-ml overnight culture) supplemented with 100 µg/ml ampicillin. The culture was induced at OD_600_ of 0.6 to 0.8 with 0.2 mM isopropyl-β-D-thiogalactopyranoside (IPTG), and continued to grow at 16°C for 24 h. The cells were harvested by centrifugation (8000 rpm, 10 min, 4°C) and resuspended in pre-cooled lysis buffer (50 mM Tris-HCl [pH 7.4], 0.9% NaCl, 1% Triton X-100, 1 mM PMSF). Then the cells were lysed by sonication on ice (300 w, working for 3 s, intermission for 8 s, 99 times), and the cell debris was removed by centrifugation (12,000 rpm, 45 min, 4°C). Later, the supernatant (containing GST-Sare0718 fusion proteins) was loaded onto a Glutathione-Sepharose4B column (GE Healthcare Life Sciences) pre-equilibrated with affinity buffer (50 mM Tris-HCl [pH 7.4], 0.9% NaCl, 1% Triton X-100). Non-specifically bound proteins were firstly removed from the resin by washing the column with affinity buffer, after which the bound fusion proteins with GST tag were recovered with elution buffer (50 mM Tris-HCl [pH 8.0], 20 mM reduced glutathione). Fractions containing the pure target protein, as determined by Coomassie blue-stained 10% SDS-PAGE gel, were combined and concentrated with ultrafiltration membrane (Millipore, MW is 30,000) before further purification by a desalting column (HiTrap™, 5 ml) using salt-exchange buffer (50 mM Tris-HCl [pH 7.5], 100 mM NaCl, 10 mM MgCl_2_, 1 mM DTT). At last, protein concentrations were determined with BCA Protein Assay Reagent (Pierce), and GST-tag integrated with Sare0718 protein was detected by western blotting analysis.

### Malachite green colorimetric assay for Sare0718 enzyme activity

#### Determination of Sare0718 substrate specificity

All 20 proteinogenic amino acids were tested to determine substrate specificity. The incubation mixtures (100 µl) contained 50 mM Tris-HCl [pH 7.5], 10 mM MgCl_2_, 10% (v/v) glycerol, 1 mM dithiothreitol (DTT), 0.5 mM amino acid, 0.5 mM ATP, 0.4 U/ml inorganic pyrophosphatase (Sigma-Aldrich, cat.no. I1643), and 0.25 µM Sare0718. Enzyme reactions were performed in 96-well plates at 25°C for 5 min, initiated by the addition of ATP. Afterwards, 100 µl malachite green reagent (0.03% (w/v) malachite, 0.2% (w/v) ammonium molybdate, and 0.05% (v/v) Triton X-100 in 0.7 M HCl) was added to stop the reaction, followed by a 10-min color development at 30°C. The absorbance at 620 nm was measured using a microplate reader (BioTek, ELx800). Each enzyme assay was performed in three different experiments, and for each amino acid, a corresponding negative control (no Sare0718 in the incubation mixture) was also included. By comparing relative activities of Sare0718 for the 20 proteinogenic amino acids, its specific substrate can be determined.

#### Selection of optimum incubation time and enzyme concentration for kinetic assay

The incubation mixtures (100 µl) contained 50 mM Tris-HCl [pH 7.5], 10 mM MgCl_2_, 10% (v/v) glycerol, 1 mM dithiothreitol (DTT), 0.5 mM alanine, 0.5 mM ATP, 0.4 U/ml inorganic pyrophosphatase, and various amounts of Sare0718 fusion protein (0.0625 µM, 0.125 µM, 0.25 µM, 0.375 µM, 0.5 µM, and 0.625 µM). Enzymatic reactions were incubated at 25°C in 96-well plates and stopped after 2 min, 5 min, 10 min, and 15 min by the addition of 100 µl malachite green reagent. After a 10-min color development at 30°C, the absorbance at 620 nm was measured using a microplate reader. A standard curve (OD620 versus PPi concentration), by which OD620 can be converted to PPi concentration, was constructed by hydrolyzing different concentrations of PPi in the incubation mixture, except that alanine, ATP, and Sare0718 proteins were omitted. Then, optimum incubation time and enzyme concentration for next kinetic assays can be chosen from enzyme concentration curve (reaction velocity versus concentration of Sare0718) and time course curve (concentration of PPi versus reaction time), respectively.

#### Determination of kinetic parameters—*K_m_*, *V_max_*, and *k_cat_*

According to the optimum incubation time and Sare0718 concentration determined above, adenylation reactions containing excessive ATP (0.5 mM) and various amounts of alanine (0.1 mM, 0.2 mM, 0.3 mM, 0.4 mM, and 0.5 mM) were done at 25°C. [PPi] was measured as described previously in this paper. The enzymatic assays were carried out in three independent replications for each alanine concentration (0.1 mM, 0.2 mM, 0.3 mM, 0.4 mM, and 0.5 mM, respectively) with a corresponding negative control (no Sare0718 in the incubation mixture). Then, the kinetic parameters of Sare0718 for L-alanine were determined by fitting the Michaelis-Menten model to the above experimental data using nonlinear regression.
